# Genetic structure of community acquired methicillin-resistant *Staphylococcus aureus* USA300

**DOI:** 10.1186/1471-2164-13-508

**Published:** 2012-09-25

**Authors:** Ryan Tewhey, Christopher R Cannavino, John AD Leake, Vikas Bansal, Eric J Topol, Ali Torkamani, John S Bradley, Nicholas J Schork

**Affiliations:** 1 Scripps Genomic Medicine, Scripps Translational Science Institute, La Jolla, CA, USA; 2 Department of Molecular and Experimental Medicine, The Scripps Research Institute, La Jolla, CA, USA; 3 Division of Biological Sciences, University of California, San Diego, La Jolla, CA, USA; 4 Department of Pediatrics, Division of Infectious Diseases, Rady Children’s Hospital San Diego, San Diego, CA, USA; 5 Department of Pediatrics, Division of Infectious Diseases, University of California, San Diego, CA, USA; 6 Department of Pediatrics, Division of Hospital Medicine, Rady Children’s Hospital San Diego, San Diego, CA, USA

## Abstract

**Background:**

Community-associated methicillin-resistant Staphylococcus aureus (CA-MRSA) is a significant bacterial pathogen that poses considerable clinical and public health challenges. The majority of the CA-MRSA disease burden consists of skin and soft tissue infections (SSTI) not associated with significant morbidity; however, CA-MRSA also causes severe, invasive infections resulting in significant morbidity and mortality. The broad range of disease severity may be influenced by bacterial genetic variation.

**Results:**

We sequenced the complete genomes of 36 CA-MRSA clinical isolates from the predominant North American community acquired clonal type USA300 (18 SSTI and 18 severe infection-associated isolates). While all 36 isolates shared remarkable genetic similarity, we found greater overall time-dependent sequence diversity among SSTI isolates. In addition, pathway analysis of non-synonymous variations revealed increased sequence diversity in the putative virulence genes of SSTI isolates.

**Conclusions:**

Here we report the first whole genome survey of diverse clinical isolates of the USA300 lineage and describe the evolution of the pathogen over time within a defined geographic area. The results demonstrate the close relatedness of clinically independent CA-MRSA isolates, which carry implications for understanding CA-MRSA epidemiology and combating its spread.

## Background

For the past 75 years, successive waves of antibiotic-resistant *Staphylococcus aureus* strains have posed significant challenges to clinicians and public health officials [1]. Beta-lactamase-mediated *S. aureus* resistance developed within a decade of widespread penicillin use, and two years after the introduction of methicillin, methicillin-resistant *S. aureus* strains (MRSA) appeared. More recently, MRSA has emerged as one of the preeminent public health threats in the United States and worldwide. In fact, all-cause *S. aureus* infections have rapidly increased during the past decade (1999, 294,570 annual U.S. cases vs. 2005, 477,927 cases) with MRSA currently accounting for >50% of staphylococcal disease [2,3]. More deaths were attributed in 2005 in the U.S. to *S. aureus* than HIV/AIDS [3]. MRSA also represents a significant economic burden with an estimated annual U.S. cost of 9 billion dollars [2]. Historically the MRSA threat consisted of hospital-acquired strains (HA-MRSA) usually affecting individuals with associated risk factors (e.g., hospitalization, multiple antibiotics).

Community-associated MRSA strains (CA-MRSA) have recently emerged as the predominant cause of MRSA disease [4]. Distinct genetic profiles suggest that CA-MRSA and HA-MRSA evolved independently. While methicillin-resistance occurs at a fitness cost to the organism in HA-MRSA strains, CA-MRSA strains have a selective advantage and generally affect previously healthy individuals [5,6]. CA-MRSA strains carry unique drug resistance genes, and widespread clinical experience suggests they may possess increased virulence compared to most HA-MRSA.

While recombination is rare among staphylococci, broad diversity exists amongst subspecies. Among CA-MRSA strains, one clonal isolate, USA300, has become predominant in the United States, representing the majority of all MRSA infections and almost all community-associated staphylococcal infection in much of the U.S.[3,4,7]. Its prevalence is also increasing in Europe [8,9]. USA300’s broad range of clinical manifestations include asymptomatic colonization, skin and soft tissue infections (SSTI), and life-threatening, severe disease (e.g. complicated pneumonia, endocarditis, osteomyelitis, and other organ-specific pathology) [10]. Given this clinical diversity, there may exist genetic differences that could serve as useful predictors of CA-MRSA-related disease phenotypes or virulence. Classical genotyping methodologies such as PFGE and MLST rely on the evaluation of highly conserved housekeeping genes representative of the vertical gene pool and resultantly provide insufficient resolution to predict disease phenotypes. Recent reports suggest that there are additional genetic components that contribute to invasiveness not captured by current typing technologies [11].

In an attempt to understand the population genetic architecture of CA-MRSA isolates and possibly resolve genomic differences relating to MRSA invasiveness, we sequenced the whole genomes of 36 CA-MRSA clinical isolates. By leveraging novel *de novo* assembly methods along with the historical time-stamps associated with the infections, we sought to determine whether there was evidence for CA-MRSA selection both across all coding genes as well as biological pathways implicated to mediate virulence.

## Results

### Clinical collection

During 2001–2006, 925 children with MRSA infections were identified at Rady Children’s Hospital in San Diego, California. To prevent the inclusion of hospital acquired strains we excluded 413 isolates (44.6%) (non-sterile sites, polymicrobial infections, chronic disease requiring frequent hospitalization, etc.). Among 512 remaining children, 41 (8%) had severe infections (among whom 36 had viable frozen isolates) and 471 (92%) had SSTI. 36 CA-MRSA SSTI-associated isolates of the same chronological distribution as that of the severe isolates were selected for genotypic comparison. The majority of the clinical diagnoses of the 36 severe CA-MRSA infections consisted of either osteoarticular infections (52.8%) or complicated pneumonias (30.6%).

There was no significant difference in patient age, gender or self-identified ethnicity between the severe infection and SSTI study groups. Compared with children with SSTI, children with severe infections had significantly longer hospital stays, longer duration of intravenous antibiotic therapy, and total duration of antibiotics (Additional file 1: Table S1).

Of all children with CA-MRSA severe infections, 3 (8.3%) children died, another 5 (13.9%) required intensive care prior to recovery, and 10 others (27.8%) were hospitalized for ≥ 10 days prior to recovery. Further, two children (5.6%), both with osteomyelitis, had long-term sequelae (chronic osteomyelitis and distal radius growth arrest as documented on follow-up) (Additional file 1: Table S2 and Additional file 1: Table S3). All children in the CA-MRSA SSTI group were hospitalized <10 days and had an uncomplicated recovery during hospitalization.

Multilocus PCR coupled to electrospray ionization-mass spectrometry (PCR/ESI-MS) genotyping revealed that all 72 severe infection and SSTI-associated CA-MRSA isolates were of a single genotype: PCR/ESI-MS genotype 1, which corresponds to PFGE USA300/USA500 and sequence type 8 designations [12]. We selected 36 isolates representing the entire collection period for whole genome sequencing (severe infections n = 18; SSTI n = 18). In addition, two replicate DNA samples from the USA300 strain FPR3757 (which has a high quality complete genome in the public database) were sequenced to serve dual functions of quality control and assistance during sequence assembly [7].

### Sequencing and data summary

We sequenced CA-MRSA isolates using the Illumina Genome Analyzer II. In order to maximize sequencing capacity, we sequenced 3 or 4 isolates per lane using nucleotide barcodes, with an average of 181 Mb of sequence per isolate. We used a custom pipeline that leveraged remapping protocols using the aligner BWA [13] against the closely related reference sequence USA300-FPR3757 as well as *de novo* assembly with Abyss [14] to successfully call polymorphisms ranging from single nucleotide variants (SNV) to large structural rearrangements. Of the core genome defined as the curated FPR3757 sequence, we sequenced to an average depth of 57-fold with 99.9% of bases across all genomes covered >5x and 93.8% >20X (Table 1).

**Table 1:** Sequencing Summary

			**All variants**					**Large events (>100 bp)**				
								**Deletions**		**Insertions**		
	**Sequenced**			**Nonubiquotus**					**Sequence**	**Mapped**	**Additional**	**Sequence**
	**Bases (Mb)**	**Coverage**	**SNV**	**SNV**	**Deletions**	**Insertions**	**Total**	**Events**	**Size**	**Events**	**Contigs**	**Size**
FPR3757 r1	155.74	56	19	-	4	1	24	0	-	0	0	-
FPR3757 r2	148.06	53	19	-	4	1	24	0	-	0	0	-
RCH_I15	164.67	59	59	35	5	2	66	0	-	0	0	-
RCH_I18	199.24	71	64	40	5	4	73	2	35.6 kb	0	0	-
RCH_I19	190.7	68	66	42	5	1	72	0	-	1	2	43.9 kb
RCH_I21	188.24	67	61	39	6	4	71	1	14 kb	0	0	-
RCH_I29	203.32	73	60	36	6	3	69	1	101 bp	1	3	41.1 kb
RCH_I33	122.77	44	59	35	6	3	68	0	-	0	1	1.8 kb
RCH_I34	168.63	60	69	45	6	3	78	0	-	0	0	-
RCH_I35	222.67	80	55	31	4	2	61	0	-	0	0	-
RCH_I36	155.12	55	79	55	11	5	95	0	-	0	0	-
RCH_I38	217.8	78	65	41	7	4	76	0	-	0	0	-
RCH_I42	125.61	45	70	46	6	7	83	0	-	0	0	-
RCH_I44	176.91	63	102	78	6	4	112	1	15.1 kb	0	0	-
RCH_I48	245.34	88	77	53	6	5	88	0	-	0	0	-
RCH_I49	191.01	68	71	47	9	3	83	0	-	0	0	-
RCH_I56	109.38	39	173^A^	149	9	4	186	0	-	1	7	43 kb
RCH_I57	126.2	45	72	48	7	3	82	0	-	2	18	73.6 kb
RCH_I58	189.15	68	71	47	6	2	79	0	-	1	1	44.2 kb
RCH_I59	180.82	65	82	58	6	6	94	1	144 bp	0	0	-
RCH_S15	181.39	65	52	28	5	1	58	1	457 bp	0	0	-
RCH_S18	121.72	43	66	42	5	2	73	0	-	0	0	-
RCH_S20	129.55	46	64	40	7	4	75	0	-	0	3	21.1 kb
RCH_S21	215.48	77	64	40	10	2	76	0	-	1	5	43.4 kb
RCH_S29	169.87	61	76	52	8	2	86	0	-	1	1	42.4 kb
RCH_S33	159.97	57	112	88	11	5	128	0	-	0	0	-
RCH_S34	207.21	74	76	52	7	3	86	1	4.2 kb	0	0	-
RCH_S35	195.87	70	61	37	4	2	67	1	-	0	0	-
RCH_S36	205.53	73	75	51	5	1	81	0	3.8 kb	1	0	44.3 kb
RCH_S38	179.7	64	89	65	7	3	99	1	766 bp	1	0	4.6 kb
RCH_S43	147.2	53	88	64	11	3	102	1	14 kb	0	0	-
RCH_S44	233.41	83	90	66	11	3	104	0	-	1	0	42.1 kb
RCH_S47	211.06	75	89	65	11	2	102	0	-	0	0	-
RCH_S49	197.15	70	68	44	4	2	74	0	-	0	0	-
RCH_S56	170.35	61	96	72	8	5	109	0	-	0	0	-
RCH_S57	185.6	66	76	52	7	3	86	0	-	1	0	42.9 kb
RCH_S59	229.36	82	86	62	9	6	101	0	-	2	2	81.9 kb
RCH_S60	202.12	72	117	93	11	2	130	1	14 kb	0	1	35.5 kb

A - 84 SNV & 1 insertion confined to a single 10 kb stretch were removed from all subsequent analysis.

**Figure 1:**
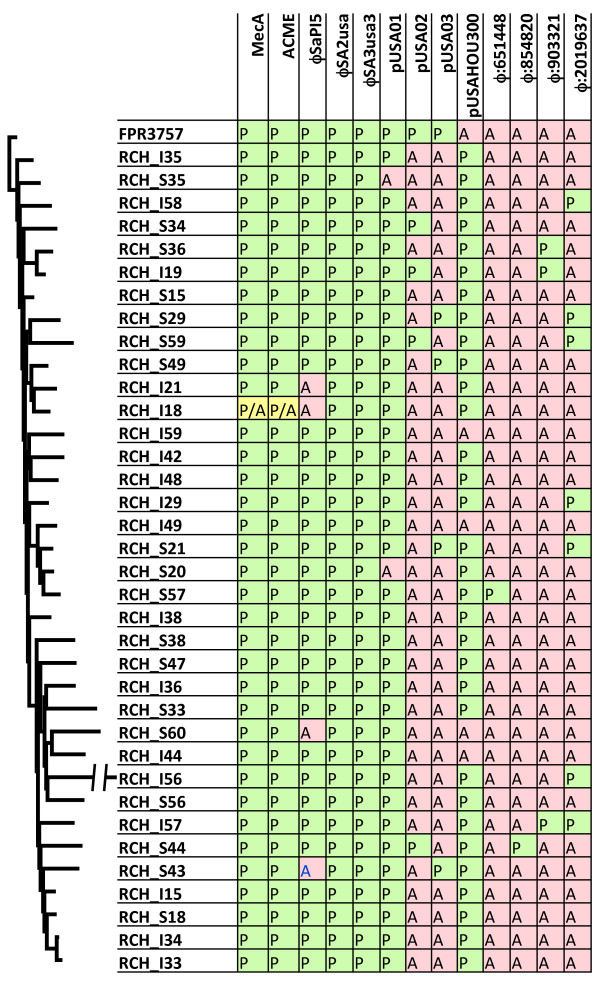
**Table exhibiting homoplasy of large insertion/deletion events.** The first 8 columns represent elements previously reported in USA300 isolates. The last 4 columns represent phage insertion sites discovered in our dataset (number denotes position in FPR3757). P and A represent presence or absence of the site respectively. The P/A for RCH I18 is a 21.6 kb deletion overlapping both the mec and ACME region but not entirely deleting either site.

In order to prevent false positive calls, we conservatively masked 3% of the genome where placing or assembling short reads could not be confidently performed. All single nucleotide events as well as large insertions and deletions that originated in the masked region were discarded. We also detected the presence of all four previously identified USA300 plasmids in our isolates. The most common combination was the presence of pUSA01 and pUSAHOU300 in 22 of the isolates (Figure 1). The remaining 14 strains represented an additional 4 combinations that is not delineated by the phylogenetic structure of the population. As noted, in addition to the 36 clinical CA-MRSA isolates, we sequenced the FPR3757 reference strain in duplicate. Perfect concor-dance was obtained between the duplicates with each sample having 24 detected variants compared to the reference sequence. Of the 24 variants, 17 were previously reported as probable errors in the reference sequence [15]. We identified 7 new variants in FPR3757, 5 of which were seen in all clinical isolates, supporting their status of true variants. The remaining two variants were visually inspected and determined as true variants. We also detected one large rearrangement in the reference sequence consisting of a 13.3 kb relocation. This rearrangement had been previously reported in a sepa-rate USA300 strain [15] and was also detected in all clinical isolates. We verified the relocation using PCR and presume it to be an error in the original assembly of the FPR3757 reference sequence.

**Figure 2:**
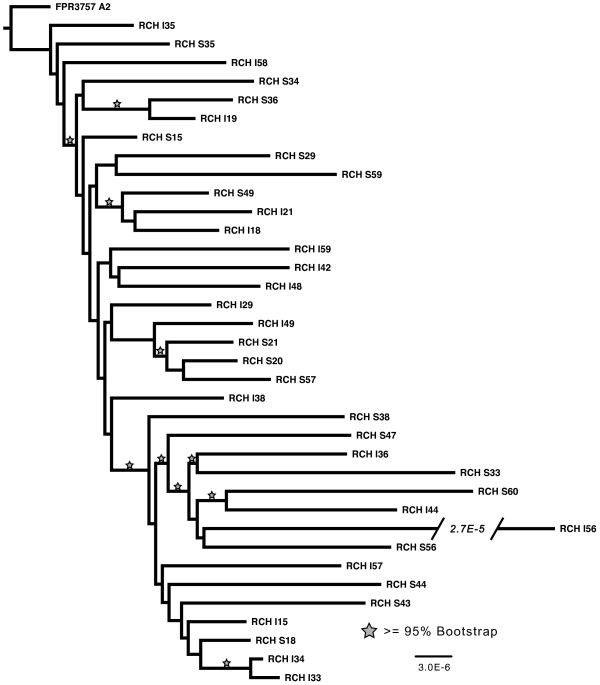
**Maximum likelihood phylogenetic tree of the 36 clinical CA-MRSA isolates and the reference strain USA300-FPR3757.** 100 bootstraps were performed and branches that carried 95% support are starred. The tree was constructed from a 2872943 bp alignment with 126 informative sites. The severe isolate designated RCH_I56 is truncated and the size of the truncation is listed on the tree.

With respect to the clinical CA-MRSA isolates, the severe and SSTI groups each carried a mean of 71 and 80 single nucleotide variants (SNV), respectively (p = 0.03, Analysis of Covariance with time as a covariate) (Table 1, Additional file 2: Figure S1). Of the 1054 SNV sites, 88% were private to a single isolate with 94% occurring at an allele frequency of less than 10% (3 or fewer isolates). We performed multiple sequence alignment on all 36 isolates as well as the resequenced FPR3757 strain and constructed a maximum-likelihood phylogenetic tree (Figure 2). There is no obvious differentiation between severe and SSTI isolates with respect to their placements on the tree. However, there are two distinct haplogroups defined by 5 variants distributed across the genome. This suggests a decisive split in the lineage of the isolates from an ancestral clone. We screened our entire collection of severe (n = 36) and SSTI (n = 36) isolates for one of the eight haplogroup-defining variants and saw a modest association between the more recently arisen haplogroup B with the less severe (SSTI) isolates (OR = 2.89, 95% CI 1.2-7.0, p = 0.03).

**Figure 3:**
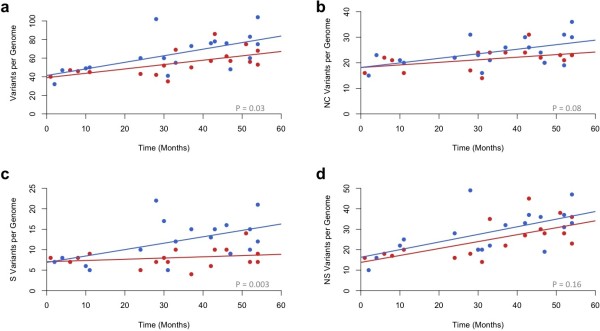
**Assessment of both total and genic mutations of the 36 clinical CA-MRSA isolates.** Number of total (**a**), noncoding (**b**) synonymous (**c**) and nonsynonymous (**d**) mutations in each of the isolates for the severe (red) and SSTI (blue) isolates are shown. Isolates are plotted according to their date of collection with the earliest isolate signifying month 1.

In both groups we observed a distinct temporal correlation supporting that the isolates are evolving away from a single common clone over time. We estimated an average mutation rate of 1.7×10^-6^ (95% confidence interval, 4.5×10^-7^ to 2.9×10^-6^) per site per year for severe isolates and 2.6×10^-6^ (95% confidence interval, 9.1×10^-7^ to 4.3×10^-6^) per site per year for SSTI isolates (Figure 3). These values are in close concordance with a previously reported estimate of 3.3×10^-6^ per site per year in hospital-acquired *S. aureus *[16]. Among SNVs, there was a transition: transversion ratio of 1.6:1 and a higher frequency of [ACGT]- > [AT] mutations, with a 1.9 fold greater occurrence, suggesting that CA-MRSA is actively introducing additional AT bias in an already GC depleted genome (Additional file 1: Table S4).

Small deletions (<100 bp) were 2-fold more frequent than small insertions (<100 bp) among the 36 clinical isolates. As expected, we found that indels were most likely to occur in homopolymer stretches, consistent with polymerase slippage during replication [17]. Large deletions were detected in ten of the isolates. The largest deletion (21.6 kb), observed in a severe isolate, included a large portion of both the SCCmec and ACME regions but retained *pbp2* (the gene responsible for methicillin resistance). The ACME region has previously been postulated to be one of the loci responsible for USA 300’s increased pathogenicity. While this locus may play a role in pathogenicity, our data suggest that the entire ACME region may not be required for severe infection. There was also a common 14 kb deletion completely removing staphylococcal pathogenicity island SaPI5 observed in 2 severe and 2 SSTI isolates. The deletion appeared on three separate bootstrapped supported branches of the phylogenetic tree, suggesting either recombination or three independent deletion events.

Large insertions (>100 bp) and additional contigs accounted for 606 kb of novel sequence across 15 isolates (6 severe and 9 SSTI isolates). For 12 isolates, we placed 14 insertions onto the core genome consisting of 269 kb of the additional sequence. Of the 14 placed insertions, 11 are large mobile genetic elements (MGEs) of prophage origin. There were four unique insertion sites for the 11 MGEs observed: 2 private appearing in only one isolate sequenced, 1 site shared by two isolates, and 1 site found in seven isolates at position 2.01 Mb on the reference sequence (Figure 1).

Of the 7 isolates sharing the large insertion located at 2.01 Mb the phylogenetic reconstruction suggests only two isolates share the insertion due to a single common ancestor with the remainder representing independent events. This insertion and the previously discussed 14 kb deletion represented two of the 11 events we detected as exhibiting homoplasy within our data (Additional file 1: Table S5). Unlike previous observations, we did not observe any convergent events with obvious implications for drug resistance [16]. This is not surprising in light of the fact that prior studies characterized HA-MRSA isolates that are presumably under different selective pressures than our pediatric CA-MRSA isolates from previously well children. Of the 9 SNVs exhibiting homoplasy, only three affected protein-coding sequence directly. However, 4 of the 9 SNVs fell within or directly adjacent to transcriptional regulators, or within putative regulatory binding sequence. This suggests that regulatory mechanisms may play an equally critical role in CA-MRSA pathogenesis as point mutations conferring functional protein-coding changes.

### Functional distribution of mutations

To assess the distribution of functional differences in our collection we annotated all non-ubiquitous va-riants of 100 bp or smaller into three distinct classes: non-synonymous coding, synonymous coding, non-coding (Additional file 1: Table S6). In line with our discovery of a greater overall abundance of variants in the SSTI isolates we saw a significantly larger number of both synonymous and non-coding variants in the SSTI isolates when compared to the severe isolates. For synonymous changes there were 219 variants in the SSTI isolates compared to 149 in the severe isolates (p = 0.003, Analysis of Covariance with time as a covariate). For non-coding changes we detected 434 changes in SSTI and 387 in the severe isolates (p = 0.08). For non-synonymous variants the trend was similar with an over abundance of variants in the SSTI isolates (SSTI = 522, severe = 455) although this difference did not pass our threshold for significance (p = 0.16). The higher proportion of synonymous variants in the SSTI isolates relative to the difference in non-synonymous variants did create an overall lower dN/dS ratio in our SSTI isolates (student’s t-test, p = 0.04). However, we are cautious of any classical interpretation of the dN/dS given the highly clonal nature of the samples and the small population size; both of which have been shown to cause fluctuation in the dN/dS calculations [18,19].

### Pathway analysis

Under the hypothesis that the SSTI isolates are evolving away from a virulent phenotype we looked for an enrichment of genes harboring unique nonsense, ns-cSNVs or deleterious frame shift mutations among the SSTI isolates in genes related to virulence. Since no single gene harbored enough variants across sufficient number of isolates for direct testing, we sought to determine if there was enrichment across all virulence-associated genes classified as being involved in “pathogenesis” or “toxin production and resistance” by the JCVI CMR database [20]. This list consisted of 94 genes encompassing 4.9% of the total coding genome. We tested against only private variation among the isolates to prevent overt bias from the few mutations that are shared. There were 335 putative functional variants found in the SSTI isolates with 27 in genes related to virulence (Table 2). This represents a 1.66 fold increase over the expected value if the placement of variation was random across the genome (binomial test p <0.01). In contrast the severe isolates carried close to the expected number at 13 va-riants out of 281, although we cannot claim a difference between the two groups given the number of isolates sampled (Chi squared of Invasive vs SSTI: p = 0.1).

**Table 2:** Variants in virulence related genes

**Gene**	**Gene ID**	**Position**	**Ref base**	**Alt base**	**Substitution**	**Invasive**	**SSTI**
immunoglobulin G binding protein A precursor	SAUSA300_0113	128554	G	T	N380K	0	1
IucC family siderophore biosynthesis protein	SAUSA300_0123	142320	G	T	S404I	1	0
sucrose-specific PTS tranporter protein	SAUSA300_0194	226384	C	T	A153V	1	0
sucrose-specific PTS tranporter protein	SAUSA300_0194	226642	G	A	G239D	0	1
choloylglycine hydrolase family protein	SAUSA300_0269	321604	C	A	P194T	0	1
essC protein	SAUSA300_0283	336391	G	A	E142K	0	1
essC protein	SAUSA300_0283	336694	A	G	I243V	1	0
superantigen-like protein 5	SAUSA300_0399	451121	C	A	A7E	1	1
superantigen-like protein	SAUSA300_0407	460962	TACAG	-----	Frameshift 108	11	10
dimethyladenosine transferase	SAUSA300_0470	530052	T	A	V258E	0	1
clumping factor A	SAUSA300_0772	859497	C	A	A117D	0	1
enterotoxin Q	SAUSA300_0801	884359	T	-	Frameshift 142	1	0
cysteine protease precursor	SAUSA300_0950	1038844	A	T	V206E	0	1
fmt protein	SAUSA300_0959	1051797	C	T	T269I	0	1
alpha-hemolysin precursor	SAUSA300_1058	1157169	G	T	T53N	0	1
putative fibronectin/fibrinogen binding protein	SAUSA300_1101	1204860	C	A	D555Y	0	2
putative enterotoxin type A	SAUSA300_1559	1708640	C	A	E78*	1	0
cell wall surface anchor family protein	SAUSA300_1702	1878567	G	A	P1968S	1	0
cell wall surface anchor family protein	SAUSA300_1702	1878783	G	C	Q1896E	0	1
cell wall surface anchor family protein	SAUSA300_1702	1878930	C	A	A1847S	0	1
cell wall surface anchor family protein	SAUSA300_1702	1880436	C	T	E1345K	0	1
cell wall surface anchor family protein	SAUSA300_1702	1880783	G	A	A1229V	0	1
serine protease SplA	SAUSA300_1758	1943333	T	C	D113G	0	1
lantibiotic epidermin biosynthesis protein EpiC	SAUSA300_1765	1950378	A	G	V209A	0	1
lantibiotic epidermin biosynthesis protein EpiB	SAUSA300_1766	1953206	T	-	Frameshift 261	0	1
leukotoxin LukD	SAUSA300_1768	1955543	C	T	V254I	1	0
leukotoxin LukE	SAUSA300_1769	1957230	T	C	K4E	0	1
Aerolysin/leukocidin family protein	SAUSA300_1975	2130674	G	A	A63V	1	0
Aerolysin/leukocidin family protein	SAUSA300_1975	2130847	T	-	Frameshift 5	0	1
hyaluronate lyase precursor	SAUSA300_2161	2338286	G	T	K65N	0	1
hyaluronate lyase precursor	SAUSA300_2161	2340008	A	T	L639F	1	0
AcrB/AcrD/AcrF family protein	SAUSA300_2213	2380381	-	T	Frameshift 1050	0	1
AcrB/AcrD/AcrF family protein	SAUSA300_2213	2382879	A	T	L218*	0	1
teicoplanin resistance associated membrane protein TcaB protein	SAUSA300_2301	2473522	C	G	W311C	0	1
IgG-binding protein SBI	SAUSA300_2364	2540728	G	A	E150K	0	1
IgG-binding protein SBI	SAUSA300_2364	2540773	C	A	Q165K	1	0
IgG-binding protein SBI	SAUSA300_2364	2540911	G	A	V211M	1	1
gamma-hemolysin component A	SAUSA300_2365	2542364	C	A	Q80K	1	0
gamma-hemolysin component C	SAUSA300_2366	2543638	A	G	I6V	0	1
putative transporter	SAUSA300_2406	2591556	A	C	M273R	2	0
clumping factor B	SAUSA300_2565	2774393	C	T	G883R	0	1

## Discussion

To address a major public health crisis that involves an infectious disease, it is essential that we track and better understand the biology of the causative pathogen. High-throughput DNA sequencing technologies provide a platform for tracking pathogens as they propagate and permit insights into virulence determinants which could be targeted pharmacologically. We sequenced the complete genomes of 36 emerging CA-MRSA isolates, half of which caused lethal or severe infections. As expected, we found evidence that all isolates of CA-MRSA are accumulating genetic variation over time, indicative of an evolution away from an ancestral clone. This rate corresponded to what has been seen in a similar study for HA-MRSA suggesting that, at least at a population wide level, these two distinct classes are evolving at a similar rate [16]. However at higher resolution these two groups likely have distinct pressures being applied to them given their differing environments. One such example is the lack of homoplasy within genes related to antimicrobial resistance. In addition we did not observe differences between the dN/dS ratios of the core and non-core portions of the genome as previously reported [21]. However, given the different population of samples and the smaller sample set that our data represents, neither discordance is troubling. We did detect that the accumulated genetic variation appears to be higher among CA-MRSA isolates causing the milder SSTI phenotypes. This distance also manifested itself by association of a distinct haplogroup, which evolved from the parental strain, with the less severe sample. Further, pathways involved in virulence are enriched among genes harboring coding variations among the clinical isolates of lesser clinical severity. Taken together, these data suggest that the invasiveness of USA 300 may be dependent, to some degree, on the genetic distance a particular clone is away from the clonal parental USA 300 strain. USA 300 was first described a little over a decade ago suggesting its emergence is recent [22,23]. The strain is a result of a two step process; first through the acquisition of the SCCmex IV complex which resulted in the clonal type USA500, followed later by the acquisition of several genes including the PVL and ACME loci [24]. Given that *Staphylococcus aureus* is a natural member of our nasal flora, as demonstrated by pervasive asymptomatic carriage [25,26], there is likely genetic pressure on the organism to maintain some degree of a commensal relationship and as a result dampen the hyper-virulent characteristic which arose from a single recombination event. This “wave” of clonality wherein an epidemic clone decreases it pathogenicity and as a result its association with infections independent of control measures has been seen in other MRSA strains [27]. Thus, the increase in genetic diversity of USA300 SSTI isolates may be an evolutionary progression away from being the hyper-virulent clonal type that USA300 represents.

The fact that we did not find any single genetic variant or set of genetic variants readily capable of distingui-shing severe- and SSTI associated CA-MRSA isolates speaks to the complexity of MRSA pathogenicity and underscores the need for more sophisticated ways to identify disease-causing CA-MRSA strains via genomic screens. While we focused our analysis to only a small number of CA-MRSA isolates displaying distinct clinical phenotypes, we are optimistic that surveying a large number of isolates (including CA-MRSA asymptomatic carrier- and MSSA-assocaited isolates) in the future will provide the power to detect evolutionary pressures at the single gene level. Of course there are two genomes at play during an infection and, as a result, a greater understanding of human genetic variation is also pivotal for the complete picture of how genetic architecture influences our interaction with microbial pathogens. It is also important to recognize that numerous non-genetic influences for virulence exist; as such to effectively investigate the genetics of pathogenicity it is critical to properly select the cases as to mitigate such effects of predisposition.

## Conclusions

Technological advances and continued decreases in the cost of sequencing are allowing the whole genome reconstruction of large collections of pathogens. These data sets offer valuable insight into the clonal population structure as well clues into the evolutionary pressures applied to the pathogen and will provide a foundation for the use of whole genome sequencing in a clinical setting. In the future, the genomic identity of individual isolates may be increasingly compared to global pathogen databases allowing the rapid identification of strains likely to cause severe disease, possess drug-resistance, and additional traits of critical utility to inform clinical decisions.

## Methods

### Isolate selection

For the five year period from January 1, 2003 to December 31, 2007, we identified two sets of children infected by CA-MRSA: (1) those with severe infections and (2) those with SSTI. Severe infections were defined as those causing invasive, potentially life-threatening clinical disease, including: complicated pneumonia (necrosis, empyema, or lung abscess); endocarditis (defined as persistent bacteremia with echocardiographic findings); deep tissue invasive abscess (mediastial, perinephric); osteoarticular infection; and pyomyositis (Additional file 1: Table S2). SSTI were defined as soft-tissue abscesses not associated with severe disease (as above) but requiring incision and drainage.To populate these two study groups, sterile-site (blood, bone, joint fluid, tracheal aspirate, pleural fluid, invasive abscess, soft-tissue abscess) MRSA isolates obtained from individual, healthy children aged 0–18 years were included. Non-sterile site (e.g. nasopharynx, stool) and polymicrobial infections were excluded. Isolates obtained from children with chronic diseases or immunocompromised states (diabetes, cystic fibrosis, neoplasm, etc.), from vascular catheters or catheter-sites and nosocomial isolates (onset after hospital admission) were also excluded.

### Demographic and clinical data

Clinical and laboratory data for all patients were collected on a standardized data collection form including: age; gender; self-identified ethnicity; infection site; length of stay (including pediatric intensive care unit stay); inpatient and discharge antibiotic treatment regimen and duration; outcome; and long-term sequelae (Additional file 1: Table S1).

### Specimen processing

Frozen MRSA specimens were thawed and inoculated onto blood-agar plates with two rounds of subculturing to ensure optimal growth. Colonies were then transferred into enriched brain/heart infusion broth media. DNA extraction was performed using a with DNeasy blood and tissue kits with lysostaphin replacing lysozyme during the initial lysis. (Qiagen, Germantown, MD).

### Illumina sequencing and preparation

For each of the 36 samples Illumina library preperation started with 5 ug of extracted DNA quantified by 260/280 (nanodrop). The standard Illumina protocol was followed through using reagents from New England Biosciences with the following exceptions: samples were sheared by either nebulization or adaptive focused acoustics (Covaris), sequencing adaptors added after a-tailing contained an addition 4 bp nucleotide barcode on the 3’ end allowing for the multiplexing of up to 4 samples per lane. After library preparation paired-end sequencing was performed for 40 cycles on each read and images were processed using Illumina pipeline 1.4. Data for each isolate can be accessed at http://sites.google.com/site/ryantewhey/home/data.

### Sequence assembly

Genome assembly utilized a mapping/denovo hybrid approach consisting of the following; Reads were first mapped using BWA [13] and default parameters to the reference FPR3757 core genome its three plasmids and the pHOU300 plasmid. Plasmids with little or no coverage were discarded. Variants and small indels with a BWA consensus score of 30 or greater and a mapping score of 10 or greater were incorporated into the sequence. We also utilized custom variant calling pipelines for further refinement of insertion/deletion detection. Mapping quality was then assessed across the genome and detected plasmids. The genome was then broken into ‘mapping contigs’ where paired-end information was discordance (outside the expected size distribution), mapping quality was high and spanning coverage was low. Separately reads were processed using the Abyss [14] assembly package using default parameters and a kmer of 23. Only assembled contigs of 1000 bp or greater were used for subsequent analysis.

Using a combination of BLAT [28] and the AMOS assembly package (http://amos.sourceforge.net), mapping and de novo contigs were merged to produce a consensus contig. In instances were the mapping and denovo contigs differed two separate contigs were produced. Reads were then mapped over the two assemblies (mapping consensus & denovo consensus) separately with BWA allowing only perfect matches. The location was then assessed at both variants for mapping quality and the variant with the greatest support via mapping was chosen.

Contigs were then scaffolded using the FPR3757 reference genome and multiple alignment was performed using the progressive Mauve engine of Mauve [29] followed by visual inspection and realignment.

### Sequence analysis

Prior to creating a phylogenetic tree all insertions and deletions were removed from the sequence and recoded as a single nucleotide transversion polyphorphism at the site of the event. A maximum likelihood tree was then constructed using RAxML [30] with 100 bootstraps on the CIPRES portal (http://www.phylo.org/). The tree was visualized and rooted using FigTree software (http://tree.bio.ed.ac.uk/software/figtree/).

For all variant analysis 24 variants were seen in all 36 clinical isolates and were removed from all subsequent analysis. In addition, 84 single nucleotide variants and 1 insertion were found clustered together within a 10 kb window of isolate I56. These variants most likely represent a single event such as recombination and not multiple unique events. Because of this they were removed from all variant analysis.

For the calculation of the dN/dS ratio we used the formula (N/n)/(S/s), where N and S are the number of nonsynonymous and synonymous mutations respectively found in the sequenced isolates, n is the total possible nonsynonymous mutations and s is the total number of possible synonymous mutations in the FPR3757 core genome. Significance of selection between groups of isolates was calculated with a two-tailed t-test. Mutation rate differences between the two isolate groups were calculated from the interaction effects of a standard linear regression. Correlation within strains to synonymous, nonsynonymous and noncoding mutations were calculated from Spearman’s non-parametric correlation coefficient.

Primary and secondary gene role categories were assigned to genes based on the J. Craig Venter Institute Comprehensive Microbial Resource annotation of *Staphylococcus aureus* USA300-FPR3757. Enrichment probabilities were calculated based on the binomial distribution, where the observed number nonsynonymous, nonsense, and frameshift mutations within a pathway was compared to the expectation due to random chance. The expected proportion of mutations occurring within a pathway is defined as the sum of the lengths of the genes belonging to the pathway over the sum of the length of all genes within the *Staphylococcus aureus* USA300-FPR3757 genome. Regions of the genome masked out during the sequence assembly and variant calling phases were not included in the pathway or whole genome summations.

### Haplotype genotyping

To genotype the larger collections of 36 severe and 36 SSTI isolates we tested one of the eight variants that differentiated between the two haplogroups. Standard PCR was performed for 35 cycles with Phusion mastermix (Finnzymes) and 100 nM of Primer A (GCAGCAATACCACCGAAAAT) and Primer B (GCGCAAGCTAGTGGGATAAG). PCR products were purified with Agencourt AMPure beads (Beckman Coulter Genomics) and subsequently digested with SpeI (New England Biolabs) overnight at 37 C. The genotype was then visually scored on a agarose gel with the uncut product corresponding to haplotype A of the reference strain FPR3757.

## Competing interests

The authors declare that they have no competing interests.

## Authors’ contributions

RT, CRC, JADL, EJT, JSB and NJS designed and supported the project. CRC identified and reviewed all clinical isolates. RT and CRC performed laboratory work. RT, VB and AT performed data analysis. RT and CRC wrote the manuscript with additions from all other authors. All authors read and approved the final manuscript.

## Supplementary Material

Additional file 1 **Table S1.**Patient characteristics of sequence isolates by study group. **Table S2**: Clinical diagnoses of included CA-MRSA severe infections. **Table S3**: Site of included CA-MRSA severe infections. **Table S4**: Nucleotide mutation catagories. **Table S5**: Variants exhibiting convergent evolution. **Table S6**: Protein Altering Single Nucleotide Variants. Click here for file

Additional file 2 **Figure S1.**Pangenomic representation of all 36 clinical isolates relative to the USA300 FPR3757 reference. Click here for file
