# Supplementary material for: Genetic structure of community acquired methicillin-resistant Staphylococcus aureus USA300
Source: BMC Genomics. 2012 Sep 25;13:508. doi: 10.1186/1471-2164-13-508 (PMC3598774; doi:10.1186/1471-2164-13-508)
Supplement: Additional file 2 — Figure S1.Pangenomic representation of all 36 clinical isolates relative to the USA300 FPR3757 reference. [file 1471-2164-13-508-S2.pdf]

Additional file 2 - Tewhey, Cannavino et al. (2012)

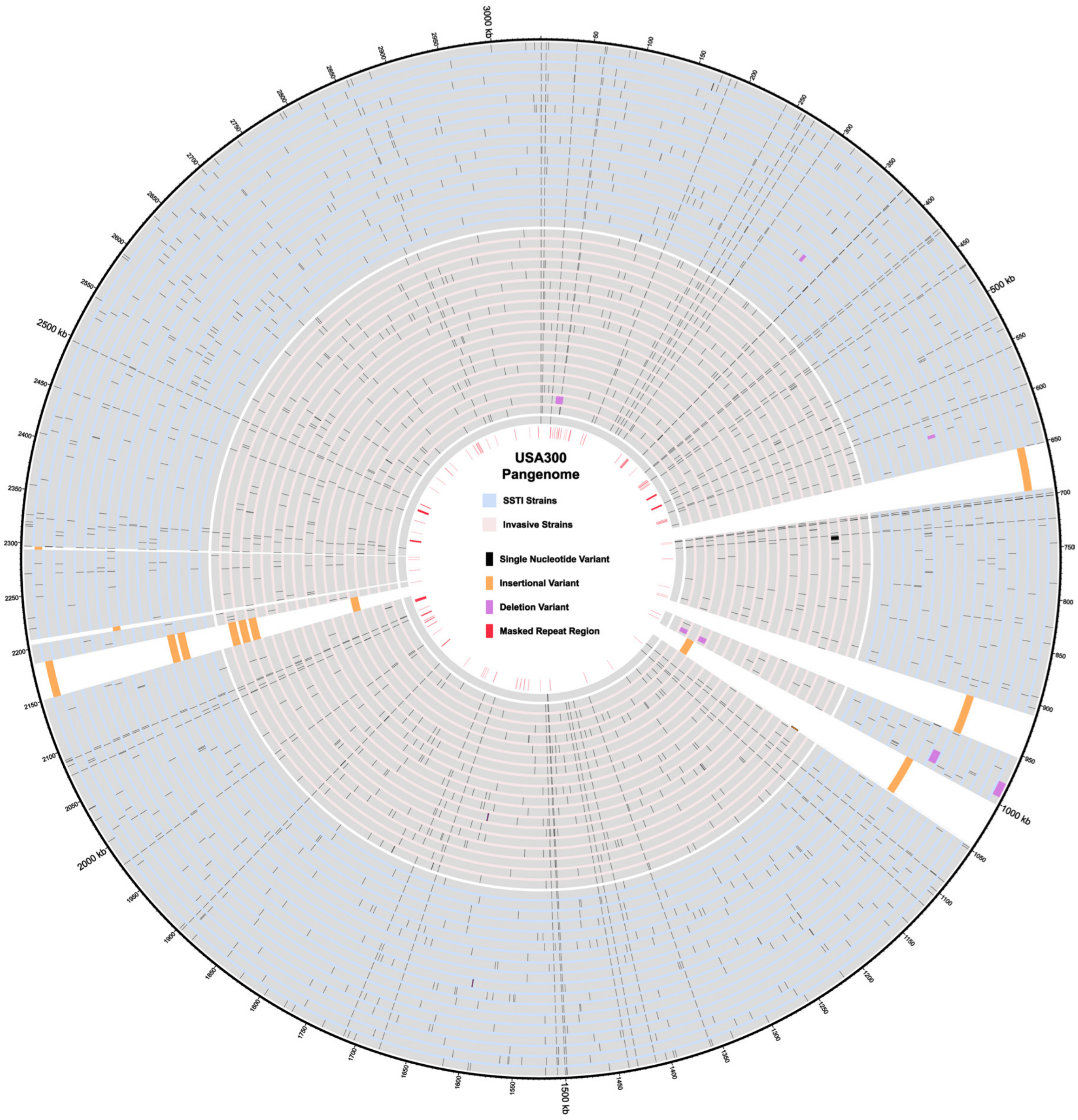

Figure S1

Pangenomic representation of the 36 CA-MRSA isolates sequenced displaying variation relative to the FPR3757 reference strain. The outer 18 tracks (highlighted in blue) represent SSTI isolates and the inner 18 tracks (highlighted in red) represent severe isolates. Insertions are marked in orange, deletions in purple and single nucleotide changes in black. The innermost track of red ticks displays repetitive regions in the FPR3757 genome.
